# Photoresponsive Gelation of Four-Armed Poly(ethylene glycol) with Photodimerizable Groups

**DOI:** 10.3390/gels8030183

**Published:** 2022-03-16

**Authors:** Masaaki Okihara, Kohei Okuma, Akifumi Kawamura, Takashi Miyata

**Affiliations:** 1 Department of Chemistry and Materials Engineering, Kansai University, 3-3-35, Yamate-cho, Suita, Osaka 564-8680, Japan; k363963@kansai-u.ac.jp (M.O.); okuma@kansai-u.ac.jp (K.O.); akifumi@kansai-u.ac.jp (A.K.); 2 Organization for Research and Development of Innovative Science and Technology, Kansai University, 3-3-35, Yamate-cho, Suita, Osaka 564-8680, Japan

**Keywords:** sol-gel phase transition, photoresponsive polymer, poly(ethylene glycol), photodimerization, tetra-PEG

## Abstract

Standard hydrogels prepared by free radical polymerization (FRP) have heterogeneous structures with a wide mesh size distribution, which affect their mechanical and separation properties. Recent research has identified four-armed poly(ethylene glycol) (tetra-PEG) as a solution to this problem. tetra-PEG gels with a homogeneous network can be prepared and applied as high-strength gels and cell-culture substrates by reacting two types of tetra-PEG with different reactive groups at the ends. In this study, we report a photoresponsive tetra-PEG that undergoes a phase transition from a sol to a gel state in response to light. tetra-PEGs containing cinnamoyl and maleimide groups at the ends of the four-armed chains were found to gel when exposed to light. The effects of polymer concentration and light irradiation time on the gelation of tetra-PEG containing photodimerization groups were investigated. The results showed that the elastic modulus of the gel increased with the increase in the light irradiation time.

## 1. Introduction

Hydrogels have attracted considerable attention in the field of biomaterials, such as cell culture substrates, drug delivery systems, and biosensors, because of their biocompatibility, flexibility, and material permeability [1]. Hydrogels are prepared by free radical polymerization (FRP), a process in which hydrophilic monomers are copolymerized with a cross-linker. Hydrogels prepared by the standard FRP method have heterogeneous structures and a wide mesh size distribution [2,3,4,5,6]. We have attempted to design hydrogels with a less heterogeneous structure by atom transfer radical polymerization (ATRP), one of the controlled radical polymerizations [7]. As a result, it was determined that hydrogels synthesized by ATRP have a less heterogeneous network structure than hydrogels synthesized by FRP. However, Sakai et al. proposed a method for forming hydrogels with a homogeneous network structure using four-armed poly(ethylene glycol) (tetra-PEG) [8,9]. When two types of tetra-PEG with amino and succinimidyl groups at the ends were mixed, PEG-based hydrogels were formed as a result of the reaction between the amino and succinimidyl groups. Various characterizations of the network structures of the resulting tetra-PEG hydrogels showed that they have homogeneous and ideal networks [9,10,11,12]. In addition, some coupling reactions, such as thiol-ene [13], azide-alkyne [14], and thioester [15] click reactions, as well as physical interactions with metal ions [16,17,18], were used to form tetra-PEG hydrogels with homogeneous network structures.

Stimuli-responsive hydrogels undergo drastic changes in properties and structures in response to external stimuli such as pH, temperature, electric field, light, biomolecules, and solvent composition. They have been investigated for use in a wide range of fields, including the medical field, as intelligent materials that exhibit unique functions, such as self-regulating drug delivery systems, artificial muscles, cell scaffolds, and sensors [19,20,21,22,23]. Takeoka et al. synthesized a four-armed poly(*N*-isopropylacrylamide) (tetra-PNIPAAm) to prepare temperature-responsive tetra-PNIPAAm hydrogels with homogeneous structures [24,25,26]. We have also designed various stimuli-responsive hydrogels using antigen-antibody complexes and carbohydrate-lectin complexes as dynamic cross-links [27,28,29,30,31,32]. For example, we synthesized tetra-PEGs terminated with biotin as biomolecule-responsive polymers capable of undergoing sol-gel phase transitions in response to specific biomolecules [33].

Various photoresponsive hydrogels have been prepared for applications in actuators and nanodevices, as light can be used as a stimulus for spatiotemporal control and remote manipulation of materials [34,35,36]. We have focused on photodimerization to design photoresponsive polymers [37]. Photodimerization occurs when a photodimerizable molecule in its ground state, such as cinnamoyl [38,39], maleimide [40,41], coumarin [42], and anthracene [43], is excited by light to form a dimer. We fabricated photoresponsive polymer films by introducing cinnamoyl groups into polydimethylsiloxane with a large free volume and photo-crosslinking only the UV-irradiated regions [44]. Photodimerization has also been utilized for forming PEG-based hydrogels for cell culture [45,46].

**Figure 1:**
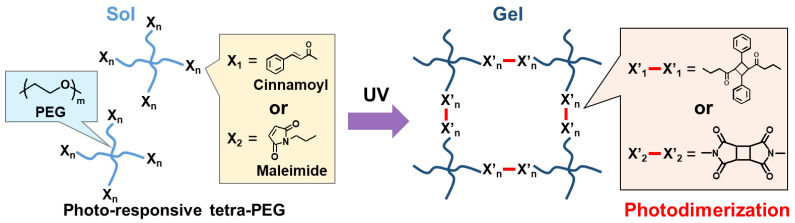
Schematic diagram of the photodimerization reaction of a photo-responsive tetra-PEG polymer.

This paper reports photoresponsive tetra-PEGs that undergo a phase transition from sol to gel states in response to light as an external stimulus. In this study, cinnamoyl and maleimide groups were introduced as photodimerization groups at the ends of tetra-PEG polymer chains. This paper focuses on the photogelation behavior of tetra-PEG with photodimerizable cinnamoyl and maleimide groups (Figure 1). Photodimerization of the cinnamoyl and maleimide groups can result in the formation of tetra-PEG hydrogels by photo-crosslinking. This paper focuses on the comparison in photodimerization and photoresponsive gelation behaviors between the tetra-PEGs with cinnamoyl and maleimide groups.

## 2. Results and Discussion

### 2.1. Synthesis of Tetra-PEG Terminated with a Cinnamoyl Group

**Scheme 1:**
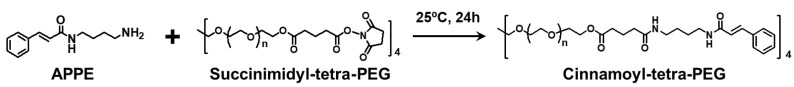
Synthetic scheme of photoresponsive tetra-PEGs terminated with cinnamoyl groups (cinnamoyl-tetra-PEG).

**Figure 2:**
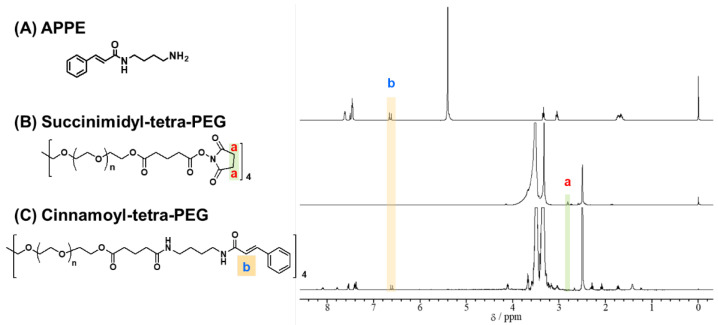
^1^H NMR spectra of (**A**) APPE (400 MHz, D_2_O, TMS, 32 times), (**B**) succinimidyl-tetra-PEG (400 MHz, DMSO-*d*, TMS, 32 times), and (**C**) cinnamoyl-tetra-PEG (400 MHz, DMSO-*d*, TMS, 32 times).

First, *N*-(4-aminobutyl)-3-phenyl-prop-2-enamide (APPE) was synthesized by introducing an amino group to cinnamoyl chloride containing a cinnamoyl group (Schemes S1–S3, Figures S1–S3). The resulting APPE was allowed to react with tetra-PEG, which has a four-armed structure and a terminal succinimidyl group (succinimidyl-tetra-PEG) to form tetra-PEG with a cinnamoyl group at the end of polymer chains (cinnamoyl-tetra-PEG) (Scheme 1). In the ^1^H NMR spectrum of the resulting cinnamoyl-tetra-PEG, the peak assigned to the cinnamoyl group was at 6.6 ppm, whereas the peak assigned to the succinimidyl group disappeared at 2.8 ppm. This shows that the cinnamoyl-tetra-PEG was successfully synthesized by the modification of succinimidyl-tetra-PEG with APPE. The ratio of the cinnamoyl group introduced into the end of four polymer chains of succinimidyl-tetra-PEG was determined to be 90.1 mol% from the peaks of PEG and the cinnamoyl group in the cinnamoyl-tetra-PEG (Figure 2).

### 2.2. Photoresponsive Gelation Behavior of Cinnamoyl-Tetra-PEG

**Figure 3:**
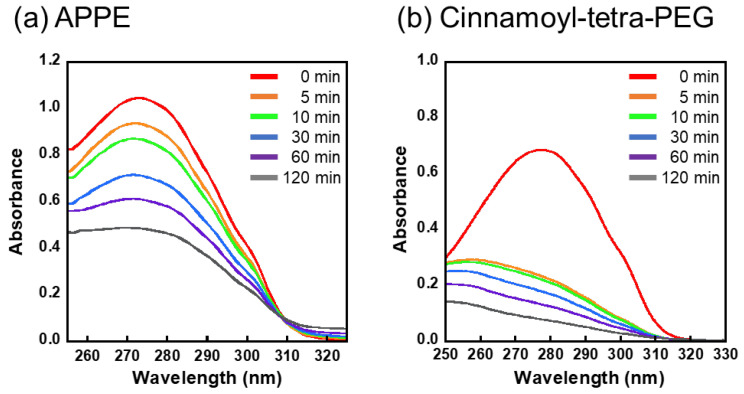
Photo-responsive behavior of cinnamoyl-tetra-PEG. (**a**) UV-Vis spectra of APPE before and after UV irradiation in DMSO at room temperature. (**b**) UV-Vis spectra of cinnamoyl-tetra-PEG before and after UV irradiation in 0.1 M pH 7.4 phosphate buffer at room temperature.

The ultraviolet (UV) and visible light (Vis) spectra of APPE and the cinnamoyl-tetra-PEG were measured after UV irradiation at a predetermined time for the evaluation of their photodimerization. The UV-Vis spectrum of APPE shows that the peak around 270 nm derived from the cinnamoyl group of APPE decreases with increasing UV irradiation time as shown in Figure 3a. This result indicates that APPE undergoes the photodimerization of the cinnamoyl groups upon UV irradiation. Similarly, the UV-Vis spectrum of cinnamoyl-tetra-PEG in Figure 3b shows that the peak derived from the cinnamoyl group of cinnamoyl-tetra-PEG decreases with increasing UV irradiation time. Unlike APPE, the absorbance of the cinnamoyl-tetra-PEG exhibits a significant drop abruptly after the UV irradiation for 5 min and then decreases gradually with time. The UV-Vis spectral measurements of APPE and cinnamoyl-tetra-PEG were performed using DMSO and a phosphate buffer solution, respectively. The hydrophobic cinnamoyl groups of the cinnamoyl-tetra-PEG are likely to be assembled in a phosphate buffer solution due to their hydrophobic interactions. This might be why the cinnamoyl-tetra-PEG exhibited a significant drop after the UV irradiation for 5 min. Thus, the difference in the assembling behavior between APPE and the cinnamoyl-tetra-PEG in solvents might influence their photodimerization behavior. From these results, we concluded that the cinnamoyl groups of the cinnamoyl-tetra-PEG undergo photodimerization in water upon UV irradiation.

**Figure 4:**
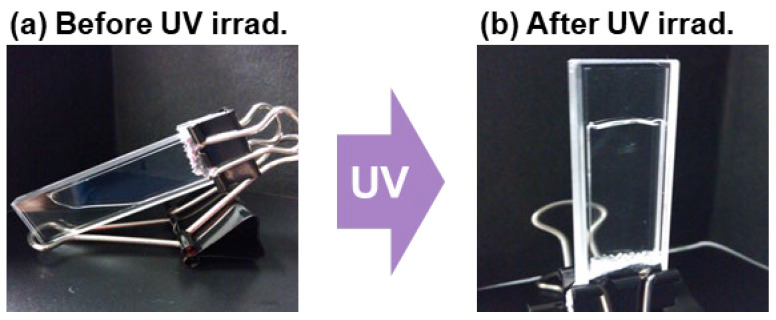
Gelation behavior of cinnamoyl-tetra-PEG under UV irradiation. Photographs of cinnamoyl-tetra-PEG before (**a**) and after (**b**) UV irradiation for 150 min.

**Figure 5:**
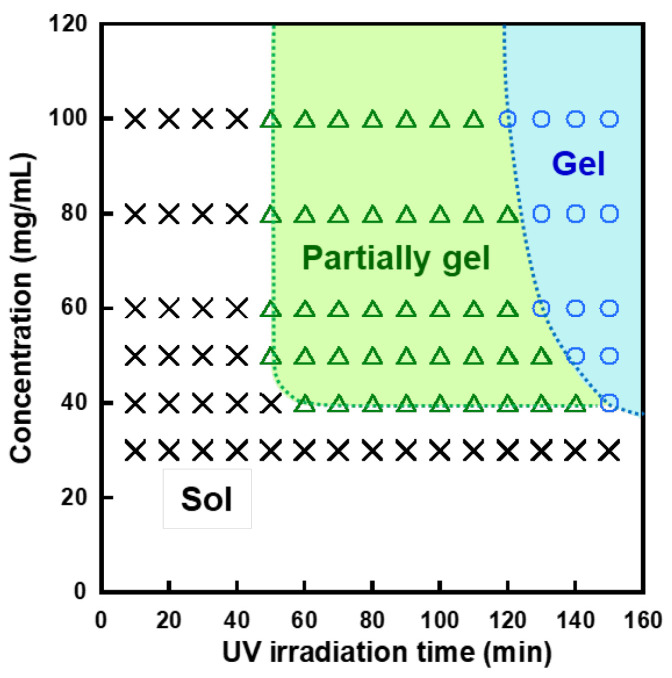
Gelation behavior of cinnamoyl-tetra-PEG under UV irradiation. Sol-gel phase diagram of cinnamoyl-tetra-PEG in 0.1 M pH 7.4 phosphate buffer after UV irradiation for 10–150 min. Symbolism: sol (×), partially gel (Δ), gel (○).

We investigated the photoresponsive gelation of an aqueous solution containing cinnamoyl-tetra-PEG at various polymer concentrations by UV irradiation. Figure 4 shows that the aqueous cinnamoyl-tetra-PEG solution undergoes a phase transition from a sol state to a gel state after UV irradiation for 150 min. This is because the cinnamoyl groups at the four-armed end of cinnamoyl-tetra-PEG form dimers by UV irradiation, followed by the formation of a three-dimensionally cross-linked network. The resultant cinnamoyl-tetra-PEG hydrogels were stable in aqueous media under ambient conditions. The phase diagram of an aqueous cinnamoyl-tetra-PEG solution after UV irradiation as a function of UV irradiation time and polymer concentration is shown in Figure 5. The phase diagram shows that a polymer concentration of more than 40 mg/mL is necessary to form tetra-PEG hydrogels by UV irradiation. An aqueous cinnamoyl-tetra-PEG solution with a higher polymer concentration undergoes sol-gel phase transition by UV irradiation for a shorter irradiation time. An aqueous cinnamoyl-tetra-PEG solution with a high polymer concentration can form a three-dimensionally cross-linked network more easily than one with a low polymer concentration when cinnamoyl groups are dimerized by UV irradiation. However, the UV irradiation time of more than 120 min was required for the gelation of an aqueous cinnamoyl-tetra-PEG solution with a polymer concentration of 100 mg/mL. Such a long UV irradiation has disadvantages in the applications of photoresponsive gelation of the cinnamoyl-tetra-PEG.

### 2.3. Photoresponsive Gelation Behavior of Tetra-PEG Terminated with Maleimide Groups

**Figure 6:**
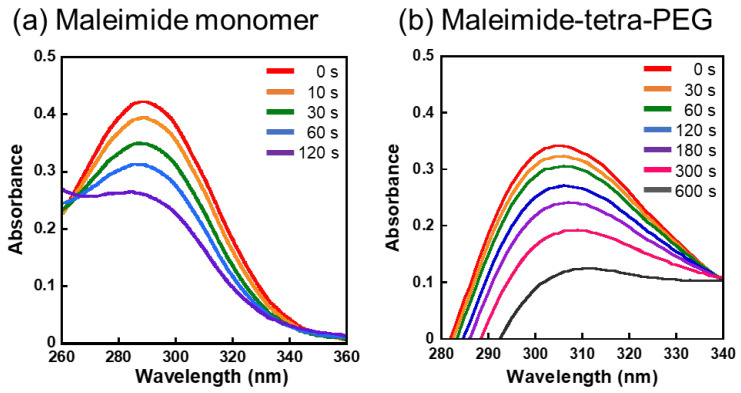
Photo-responsive behavior of maleimide-tetra-PEG. (**a**) UV-Vis spectra of maleimide monomer before and after UV irradiation in DMSO at room temperature. (**b**) UV-Vis spectra of maleimide-tetra-PEG before and after UV irradiation in 0.1 M pH 7.4 phosphate buffer at room temperature.

The photoresponsive gelation of an aqueous cinnamoyl-tetra-PEG solution requires more than 120 min of UV irradiation. Therefore, a photoresponsive tetra-PEG that undergoes sol-gel phase transition by UV irradiation for a shorter time period is required for practical use. Unlike cinnamic acid, the photodimerization of maleimide proceeds through 1,4-biradicals, showing that maleimide groups undergo photodimerization for a shorter period of time than cinnamoyl groups [40,47], as shown in Figure S4. To optimize the photoresponsive gelation of cinnamoyl-tetra-PEG, we investigated the gelation behavior of tetra-PEG with a maleimide group at the four-armed end (maleimide-tetra-PEG). The UV-Vis spectra of aqueous solutions of *N*-(2-aminoethyl)maleimide trifluoroacetate salt and maleimide-tetra-PEG after UV irradiation is shown in Figure 6. In both the aqueous solutions, the peak at around 290 nm derived from the maleimide group decreased with increasing UV irradiation time. This indicates that the maleimide groups of the maleimide-tetra-PEG undergo photodimerization by UV irradiation. Interestingly, the photodimerization of the cinnamoyl-tetra-PEG proceeds on the order of minutes, as shown in Figure 3, whereas that of maleimide-tetra-PEG proceeds on the order of seconds, as shown in Figure 6. This may be because, unlike the cinnamoyl group, the photodimerization of the maleimide group in the maleimide-tetra-PEG occurs through an active radical [40,47].

**Figure 7:**
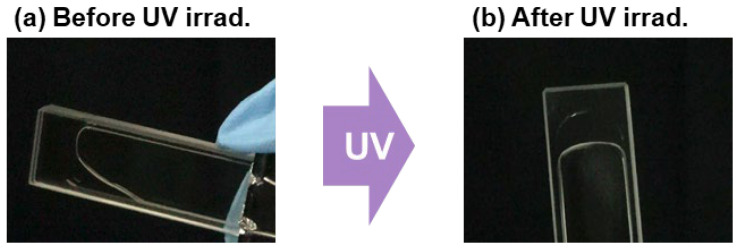
Gelation behavior of maleimide-tetra-PEG under UV irradiation. Photographs of maleimide-tetra-PEG before (**a**) and after (**b**) UV irradiation for 2 min.

**Figure 8:**
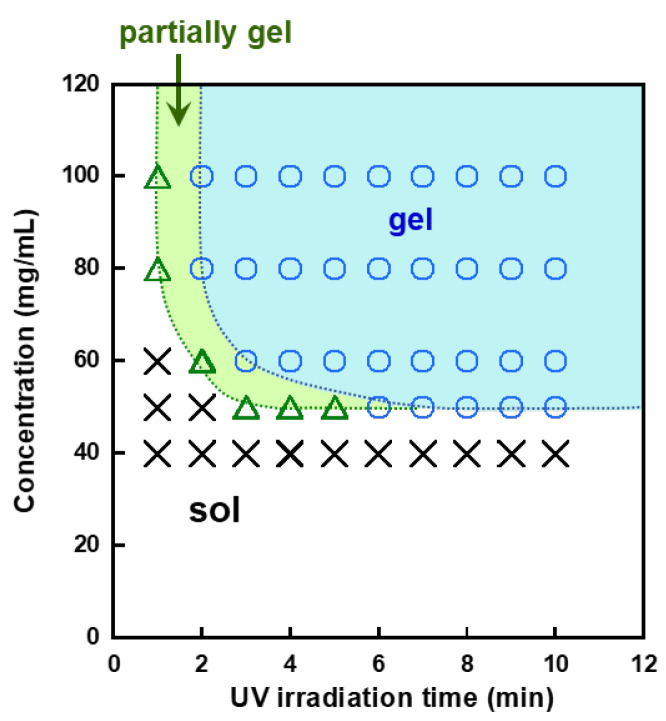
Gelation behavior of maleimide-tetra-PEG under UV irradiation. Sol-gel phase diagram of maleimide-tetra-PEG in 0.1 M pH 7.4 phosphate buffer after UV irradiation for 2–10 min. Symbolism: sol (×), partially gel (Δ), gel (○).

The sol-gel phase transition of an aqueous solution containing the maleimide-tetra-PEG was investigated during UV irradiation. An aqueous maleimide-tetra-PEG solution with a polymer concentration of 100 mg/mL changed from a sol state to a gel state by UV irradiation for only 2 min (Movie S1), as shown in Figure 7. The resultant maleimide-tetra-PEG hydrogels were stable in aqueous media under ambient conditions. A phase diagram of an aqueous maleimide-tetra-PEG solution with various polymer concentrations after UV irradiation for various irradiation times is shown in Figure 8. Similar to the photoresponsive gelation of the cinnamoyl-tetra-PEG shown in Figure 5, an aqueous maleimide-tetra-PEG solution with a higher polymer concentration undergoes sol-gel phase transition by UV irradiation for a shorter irradiation time. Notably, although the gelation of an aqueous cinnamoyl-tetra-PEG solution with a polymer concentration of 100 mg/mL required UV irradiation for 120 min, as shown in Figure 5, that of an aqueous maleimide-tetra-PEG solution with the same polymer concentration was induced by UV irradiation for only 2 min. Namely, the UV irradiation time for the gelation of the aqueous maleimide-tetra-PEG solution was reduced to 1/60. This may be attributed to the different mechanisms in the photodimerization between the cinnamoyl and maleimide groups. The reduction in UV irradiation time for the gelation of an aqueous maleimide-tetra-PEG solution can also be explained by the quicker photodimerization of maleimide groups than that of cinnamoyl groups, as shown in Figure 3 and Figure 6. When cinnamoyl groups are exposed to UV, the cis-trans isomerization occurs before the photodimerization reaction, and the photodimerization proceeds afterwards [48]. However, the maleimide groups do not undergo a side reaction and dimerize through the highly reactive 1,4-biradical under UV irradiation [40,47], as shown in Figure S4. As a result, the reaction rate in the photodimerization of the maleimide groups becomes significantly faster than that of cinnamoyl groups. As a result of its gelation upon UV irradiation for a few minutes, the maleimide-tetra-PEG is suitable as a photoresponsive sol-gel phase transition polymer for practical applications such as cell culture substrates.

### 2.4. Effect of UV Irradiation Conditions on the Viscoelastic Behavior of Maleimide-Tetra-PEG Hydrogels

**Figure 9:**
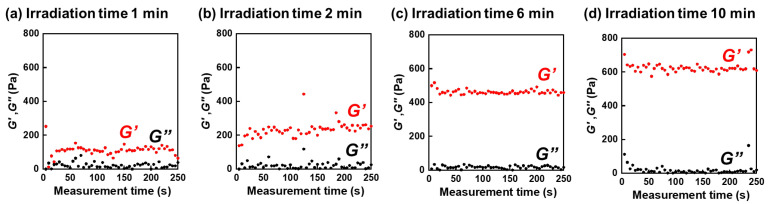
Elastic modulus changes of maleimide-tetra-PEG gel under different UV irradiation times. Storage modulus (*G*′) and loss modulus (*G*″) of maleimide-tetra-PEG gels prepared by irradiating maleimide-tetra-PEG aqueous solution with UV light at different irradiation times of 1 (**a**), 2 (**b**), 6 (**c**), and 10 (**d**) minutes, measured under time-dependent conditions of constant frequency.

**Figure 10:**
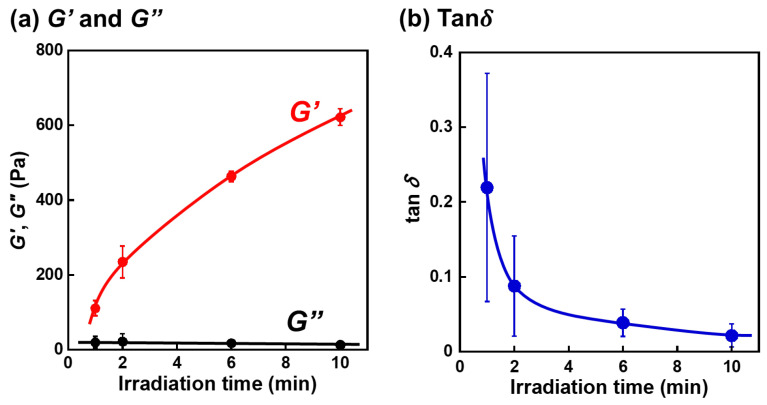
Effect of UV irradiation time on elastic modulus (*G*′ and *G*″) (**a**) and tan*δ* (**b**) calculated by averaging the elastic modulus changes of each maleimide-tetra-PEG gel in Figure 9.

The photoresponsive sol-gel transition of maleimide-tetra-PEG by UV irradiation for only a few minutes enables the formation of tetra-PEG hydrogels, which is likely to be similar to the tetra-PEG hydrogels reported previously [8,9,10]. Such tetra-PEG hydrogels are useful in cell culture and other medical applications. From a mechanobiological point of view, the mechanical properties of hydrogels used for cell culture are important factors in regulating cell behaviors such as adhesion, proliferation, and differentiation. Therefore, we performed rheological measurements of the hydrogels formed from maleimide-tetra-PEG by UV irradiation. The storage modulus (*G*′) and loss modulus (*G*″) of the maleimide-tetra-PEG hydrogels prepared by exposing aqueous solutions of maleimide-tetra-PEG to UV light are shown in Figure 9. After the UV irradiation for more than 1 min, *G*′ of the aqueous solution of maleimide-tetra-PEG became larger than *G*″. This indicates that the aqueous maleimide-tetra-PEG solution changes from a sol state to a gel state by the UV irradiation of only 1 min, unlike the photoresponsive sol-gel transition of cinnamoyl-tetra-PEG shown in Figure 5. A change in the *G*′, *G*″, and tan*δ* (G″/G′) of the aqueous solution of the maleimide-tetra-PEG as a function of the UV irradiation time was shown in Figure 10. The *G*″ of the aqueous solution of maleimide-tetra-PEG remained constant as the UV irradiation time increased, but the *G*′ increased monotonically. The monotonic increase in *G*′ observed after UV irradiation is due to an increase in the crosslink density of the resultant PEG networks caused by photodimerization of maleimide groups. The tan*δ* of less than one in Figure 10b means that the aqueous solution becomes in the gel state after the UV irradiation for a few minutes. A decrease in tan*δ* with increasing the UV irradiation time indicates that elastic characteristics become more predominant than their viscous characteristics in the viscoelastic behavior of the resultant tetra-PEG hydrogels [49,50]. As a result, it is clear that UV irradiating the maleimide-tetra-PEG undergoes sol-gel transition for only a few minutes because the photodimerization of the maleimide groups at their four-armed ends induces the formation of three-dimensional PEG networks. In addition, the UV irradiation time can be used to control the elastic modulus of the resulting maleimide-tetra-PEG hydrogels. This means that tetra-PEG hydrogels with spatiotemporally tuned elastic moduli can be easily prepared by exposing an aqueous solution of maleimide-tetra-PEG to UV light for only a few minutes. Medical applications of the photoresponsive sol-gel transition of maleimide-tetra-PEG require a detailed understanding of the structural factors that affect the mechanical properties required for cell regulation. However, the knowledge gained from the successful formation of tetra-PEG hydrogels by light irradiation for a short time not only provides a basis for the development of synthetic scaffolds with tunable stiffness for cell culture but also provides additional insight into the structural design of photoresponsive polymer networks.

## 3. Conclusions

In summary, we investigated the photoresponsive gelation behavior of a four-armed PEG terminated with a cinnamoyl or maleimide group as a photodimerizable group (cinnamoyl or maleimide-tetra-PEG). An aqueous solution of cinnamoyl-tetra-PEG with a polymer concentration of 100 mg/mL gelated after 120 min of UV irradiation. However, an aqueous solution of maleimide-tetra-PEG with a polymer concentration of 100 mg/mL underwent a sol-gel phase transition after only 2 min of UV irradiation. Photoresponsive gelation of an aqueous solution of maleimide-tetra-PEG in a much shorter time than that of cinnamoyl-tetra-PEG is attributed to the quicker photodimerization of maleimide groups than cinnamoyl groups. Rheological experiments showed that the elastic modulus of the hydrogels formed from maleimide-tetra-PEG gel can be controlled by the UV irradiation time. As a result, tetra-PEG hydrogels with spatiotemporally tuned elastic moduli were easily prepared by exposing an aqueous solution of maleimide-tetra-PEG to UV light for only a few minutes. From the point of mechanobiological view, the maleimide-tetra-PEG and the resultant tetra-PEG hydrogels are anticipated to be applied as photoresponsive scaffolds and biomaterials for regulating cells.

## 4. Materials and Methods

### 4.1. Materials

1,4-Butanediamine, di-*tert*-butyl di-carbonate, chloroform, dichloromethane, triethylamine, sodium hydroxide, ethanol, diethyl ether, sodium chloride, *N*,*N*-dimethylformamide (DMF), hydrochloric acid, dimethyl sulfoxide (DMSO), and *N*-(2-Aminoethyl)maleimide trifluoroacetate salt were purchased from FUJIFILM Wako Pure Chemical Corporation (Osaka, Japan) and used as received. Cinnamoyl chloride was purchased from Tokyo Chemical Industry Co., Ltd. (Tokyo, Japan) and used as received. Succinimidyl-tetra-PEG (pentaerythritol tetra (succinimidyloxyglutaryl) polyoxyethylene), maleimide-tetra-PEG (*N*-(2-aminoethyl)maleimide trifluoroacetate salt, and Pentaerythritol tetra {[3-(3-maleimido-1-oxopropyl)amino]propyl} polyoxyethylene) were purchased from NOF Corporation (Tokyo, Japan) and used as received. All aqueous solutions were prepared with ultrapure water (Milli-Q, 18.2 MΩ·cm). Other solvents and reagents of analytical grade were obtained from commercial sources and were used without further purification.

### 4.2. Synthesis of Tert-butyl 4-aminobutylcarbamate and Tert-butyl N-[4-(3-phenyl-prop-2-enoylamino)-butyl]carbamate

A solution of 1,4-butanediamine (10.0 g, 113 mmol) in 119 mL of chloroform was stirred for 16 h under an ice bath while a solution of di-*tert*-butyl di-carbonate (2.74 g, 12.6 mmol) in 72.6 mL of chloroform was slowly added dropwise (Scheme S1). After stirring, the solution was extracted three times with 175 mL of ultrapure water. After extraction, *tert*-butyl 4-aminobutylcarbamate (yield: 1.56 g, 71.5%) was produced by dehydration and removal of the solvent. Then, the produced *tert*-butyl 4-aminobutylcarbamate (1.56 g, 8.38 mmol) was dissolved in 17.5 mL of dichloromethane. To this solution was added 1.99 mL of triethylamine, and a solution of cinnamoyl chloride (2.03 g, 12.1 mmol) dissolved in 8.49 mL of dichloromethane was mixed dropwise under an ice bath for two hours (Scheme S2). After stirring, the solution was washed three times with 42.5 mL of ultrapure water and subsequently extracted three times with 42.5 mL of aqueous sodium hydroxide solution. After extraction, the solvent was removed by dehydration to produce yellow solid *tert*-butyl *N*-[4-(3-phenyl-prop-2-enoylamino)-butyl]carbamate (yield: 2.05 g, 77.0%).

### 4.3. Synthesis of N-(4-aminobutyl)-3-phenyl-prop-2-enamide (APPE)

*tert*-Butyl *N*-[4-(3-phenyl-prop-2-enoylamino)-butyl]carbamate (2.05 g, 6.43 mmol) was dissolved in 16.5 mL of ethanol. To this mixture, 6 mL of 2 M hydrochloric acid was added, and the mixture was stirred at 40 °C for three hours (Scheme S3). Then, 10 mL of diethyl ether was added, and the mixture was extracted three times with 15 mL of a saturated aqueous sodium chloride solution. After extraction, an aqueous sodium hydroxide solution was added to the resultant aqueous phase, and the mixture was extracted three times with 75 mL of dichloromethane. The synthesis of APPE was confirmed by ^1^H NMR measurement using an NMR system (AL400: JEOL Ltd., Tokyo, Japan) with 10 mg/mL of the product produce by the above procedure dissolved in DMSO-*d*.

### 4.4. Synthesis of Cinnamoyl-tetra-PEG

Succinimidyl-tetra-PEG (53.2 mg, 1.29 μmol) was dissolved in 1 mL of DMF. APPE (2.27 mg, 10.4 μmol) was dissolved in 16.9 μL of DMF. After mixing the succinimidyl-tetra-PEG and APPE solutions, the mixture was incubated at 25 °C for 24 h (Scheme 1). The precipitates were then dissolved in 2.0 mL of 0.1 M HCl and dialyzed for 24 h using a dialysis membrane (12 kD to 15 kD). For the synthesis of cinnamoyl-tetra-PEG, the product produced by the above procedure was dissolved in DMSO-*d* at 10 mg/mL, and NMR spectroscopy (AL400: JEOL Ltd., Tokyo, Japan) was performed.

### 4.5. UV-Vis Spectral Measurements of Cinnamoyl-tetra-PEG and Maleimide-tetra-PEG

APPE was dissolved in DMSO at a concentration of 0.85 mg/mL, whereas cinnamoyl-tetra-PEG, *N*-(2-Aminoethyl)maleimide trifluoroacetate salt, and maleimide-tetra-PEG were dissolved in a phosphate buffer solution (0.1 M, pH 7.4) at a concentration of 3 mg/mL. After the resultant solutions were exposed to UV light for various lengths of time with a high-pressure mercury lamp (Optical Modulex SX-UI502HQ: Ushio Inc., Tokyo, Japan), UV-Vis spectrophotometers (UV-2550: Shimadzu Co., Kyoto, Japan) were used to measure their UV-Vis spectra.

### 4.6. Gelation Behavior of Cinnamoyl-tetra-PEG and Maleimide-tetra-PEG

Cinnamoyl- and maleimide-tetra-PEG were dissolved in a phosphate buffer solution (0.1 M, pH 7.4) at various concentrations. After the aqueous solutions of cinnamoyl- and maleimide-tetra-PEG were exposed to UV light for a predetermined period using a high-pressure mercury lamp (Optical Modulex SX-UI502HQ: Ushio Inc., Tokyo, Japan), their gelation behavior was evaluated by the gradient method in which the flowability of the solution was observed by turning the container upside down. When the sample did not demonstrate the flowability at all after the container was turned upside down and kept for 30 min, the sample was defined as “gel”. On the other hand, when the sample exhibited the flowability but its part was in a gel state, the sample was assigned as “partial gel”. When the sample exhibited the flowability completely without any partial gels, the sample was assigned as “sol”. The effect of the polymer concentrations, cinnamoyl-tetra-PEG and maleimide-tetra-PEG, and the UV irradiation time on the gelation of the solution was summarized in phase diagrams in Figure 5 and Figure 8, respectively.

### 4.7. Viscoelasticity Measurement of Maleimide-tetra-PEG

Maleimide-tetra-PEG was dissolved in a phosphate buffer solution (0.1 M, pH 7.4) with a polymer concentration of 80 mg/mL. The aqueous solution of maleimide-tetra-PEG in a quartz glass mold was exposed to UV light for a predetermined period using a high-pressure mercury lamp (Optical Modulex SX-UI502HQ: Ushio Inc., Tokyo, Japan). The rheological measurements were performed under the condition with a constant strain of 0.1% and a constant frequency of 1 Hz at 25 °C using a RheoStress 300 (Anton Paar, Japan, MCR102) arranged in parallel-plate geometry. Storage modulus (*G*′) and loss modulus (*G*″) of the solutions or hydrogels were evaluated under time-dependent conditions with a constant frequency.

## Data Availability

Not applicable.

## Supplementary Materials

The following supporting information can be downloaded at: https://www.mdpi.com/article/10.3390/gels8030183/s1, Scheme S1: Synthesis of tert-1,4-aminobutylcarbamate; Scheme S2: Synthesis of tert-butyl-N-[4-(3-phenyl-prop-2-enoylamino)-butyl] carbamate; Scheme S3: Synthesis of N-(4-aminobutyl)-3-phenyl-prop-2-enamide (APPE); Figure S1: 1H NMR spectrum of tert-1,4-aminobutylcarbamate (400 MHz, CDCl3, 32 times); Figure S2: 1H NMR spectrum of tert-butyl-N-[4-(3-phenyl-prop-2-enoylamino)-butyl] carbamate (400 MHz, CDCl3, 32 times); Figure S3: 1H NMR spectrum of N-(4-aminobutyl)-3-phenyl-prop-2-enamide (APPE) (400 MHz, D2O, TMS, 32 times); Figure S4: Schematic addressing the reaction mechanism for the photodimerization of the maleimide groups; Movie S1: The sol-gel phase transition of an aqueous solution containing the maleimide-tetra-PEG by UV irradiation.
