# Supplementary material for: Photoresponsive Gelation of Four-Armed Poly(ethylene glycol) with Photodimerizable Groups
Source: Gels. 2022 Mar 16;8(3):183. doi: 10.3390/gels8030183 (PMC8950757; doi:10.3390/gels8030183)
Supplement: Supplementary file 1 [file gels-08-00183-s001.zip › gels-1620721-supplementary.pdf]

Supporting Information

Photoresponsive Gelation of Four-Armed Poly(ethylene glycol) with Photodimerizable Groups

Masaaki Okihara [1], Kohei Okuma [1], Akifumi Kawamura[1, 2] and Takashi Miyata [1, 2,]*

[1]Department of Chemistry and Materials Engineering, Kansai University, 3-3-35, Yamate-cho, Suita, Osaka 564-8680, Japan

[2]Organization for Research and Development of Innovative Science and Technology, Kansai University, 3-3-35, Yamate-cho, Suita, Osaka 564-8680, Japan

*Correspondence: e-mail: tmiyata@kansai-u.ac.jp

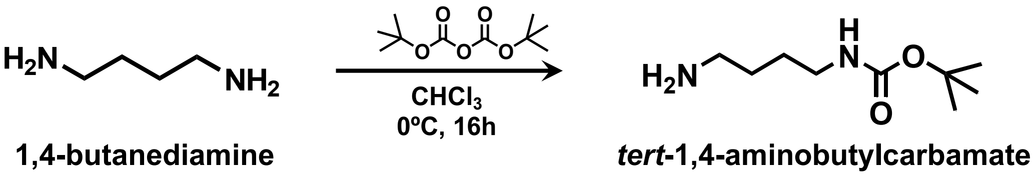

Scheme S1. Synthesis of *tert*-1,4-aminobutylcarbamate.

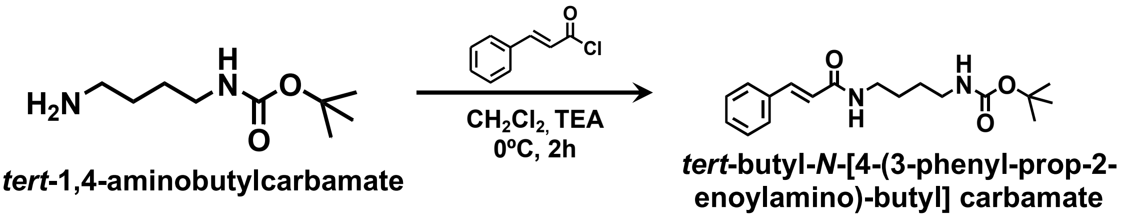

Scheme S2. Synthesis of *tert*-butyl-N-[4-(3-phenyl-prop-2-enoylamino)-butyl] carbamate.

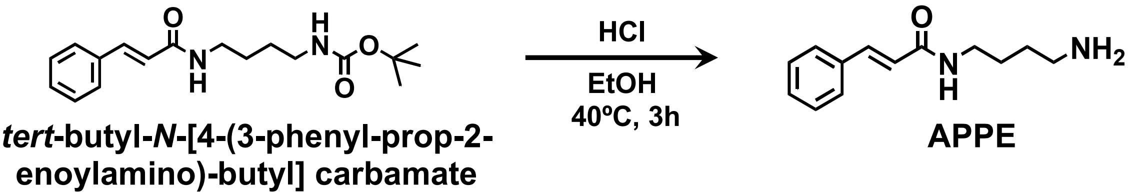

Scheme S3. Synthesis of *N*-(4-aminobutyl)-3-phenyl-prop-2-enamide (APPE).

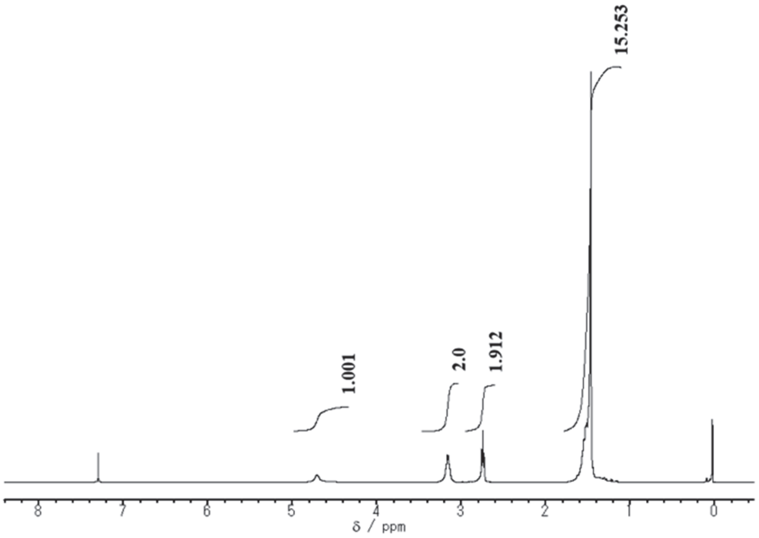

Figure S1. ^{1}H NMR spectrum of *tert*-1,4-aminobutylcarbamate (400 MHz, $CDCl_3$, 32 times).

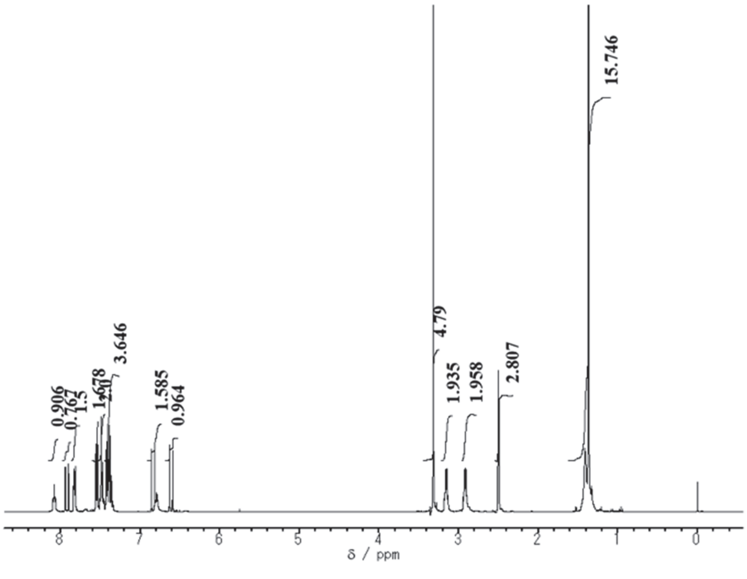

Figure S2. ^{1}H NMR spectrum of *tert*-butyl-N-[4-(3-phenyl-prop-2-enoylamino)-butyl] carbamate (400 MHz, $CDCl_3$, 32 times).

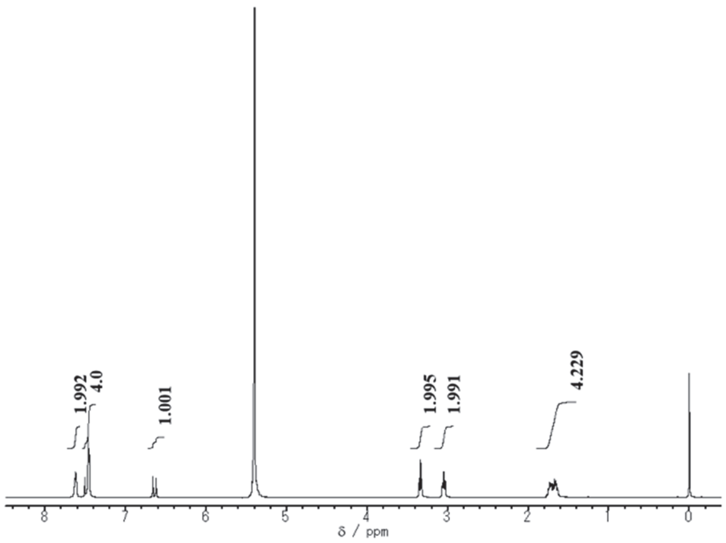

Figure S3. ^{1}H NMR spectrum of *N*-(4-aminobutyl)-3-phenyl-prop-2-enamide (APPE) (400 MHz, D_2O, TMS, 32 times).

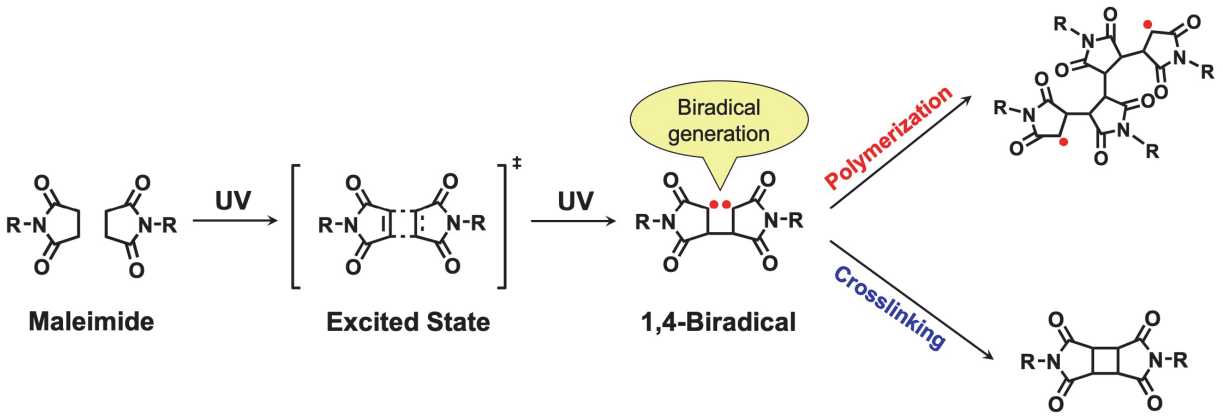

Figure S4. Reaction mechanism for the photodimerization of the maleimide groups of maleimide-tetra-PEG.
